# Coronary Computed Tomography Angiography (CTA) in the Diagnostic Triage of Patients Undergoing Liver Transplantation: A Long-term Outcome Study

**DOI:** 10.1016/j.jceh.2025.103200

**Published:** 2025-10-07

**Authors:** Pietro G. Lacaita, Armin Finkenstedt, Thomas Senoner, Heinz Zoller, Guy Friedrich, Mathias Pamminger, Yannick Scharll, Gerlig Widmann, Gudrun M. Feuchtner

**Affiliations:** ∗ Innsbruck Medical University, Department Radiology, Austria; † Innsbruck Medical University, Department Internal Medicine, Emergency Medicine, Austria; ‡ Innsbruck Medical University, Department Anaesthesia and Intensive Care, Austria; § Innsbruck Medical University, Department Internal Medicine II, Gastroenterology, Austria; ‖ Innsbruck Medical University, Department Internal Medicine III, Cardiology, Austria

**Keywords:** computed tomography angiography, liver transplantation, end-stage liver disease, cardiovascular risk, coronary artery disease

## Abstract

**Background:**

Cardiovascular risk stratification is crucial in patients with end-stage liver disease (ESLD) yet the optimal noninvasive strategy remains debated**.** Our study aimed to assess the prognostic value of coronary computed tomography angiography (CTA) and coronary artery calcium (CAC) in patients undergoing orthotopic liver transplantation (LT).

**Methods:**

Patients with ESLD scheduled for LT referred to coronary CTA and the CACscore were included. The primary endpoint was all-cause mortality and the secondary endpoint was myocardial infarction (MI).

**Results:**

Four hundred fifty-eight patients for pre-LT risk stratification were enrolled with 270 LT recipients (79.3% male; mean age 61 ± 8.5 years) finally being included. The mean follow-up was 7.5 ± 3.1 years, range: 2–13. Among 248 patients undergoing CTA, the majority (n = 173, 69.8%) had coronary artery disease (CAD) by CTA (Coronary Artery Disease—Reporting and Data System[CAD-RADS] 1–5), and n = 75 (30.2%) had no CAD. Stenosis severity was minimal-to-mild (<50%) in 112 (45.1%), intermediate (50–70%) in 44 (17.7%), and severe (>70%) in 17 (6.5%) patients. The all-cause mortality rate was 46 (17.0%) (n = 3 cardiovascular). Stenosis severity (CAD-RADS) was associated with mortality (Kaplan–Meier analysis, *P* < 0.001). On multivariate Cox regression, total plaque burden were associated with all-cause mortality (hazard ratio [HR]: 1.1, *P* = 0.034; 95% confidence interval [CI]: 0.649–0.983 and HR: 1.1, *P* = 0.029; 95% CI: 1.0–1.6), while the CAC score was not. Six acute myocardial infarctions (MIs, 3 ST-elevation MI [STEMI] and 3 non-STEMI) occurred, none of them (0%) in patients with CAD-RADS 0–1 and 100% CAD-RADS 2–4.

**Conclusion:**

Coronary CTA is a valuable tool for pre-LT cardiovascular risk assessment. Patients with no or minimal CAD on CTA have an excellent prognosis regarding survival.

**Clinical relevance statement:**

Coronary CTA enables refined risk stratification in patients undergoing liver transplantation by assessing stenosis severity and plaque burden.

## Graphical abstract

**Figure:**
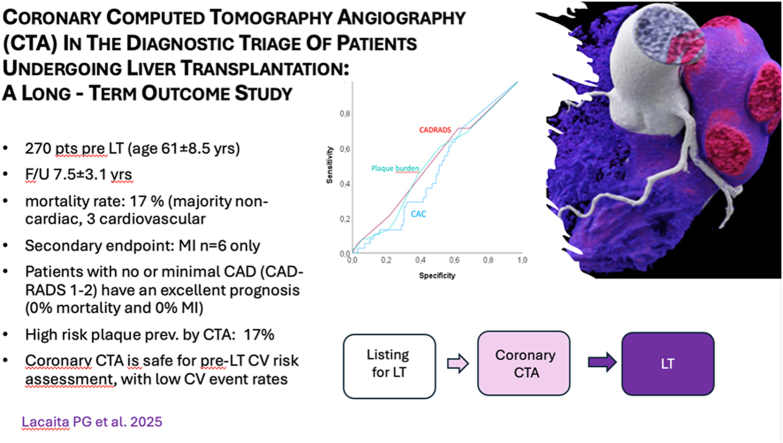

Liver transplantation (LT) remains the only curative therapy for patients with advanced liver disease, with 5- to 10-year survival rates exceeding 60% as reported by both European and American liver transplant registries.^1^^,^^2^

Cardiovascular complications are the third leading cause of death and postoperative morbidity after LT—preceded only by infections and graft rejection—with reported incidence rates ranging from 25% to 70%.^3^

Moreover, LT recipients are at increased risk of coronary artery disease (CAD),^4^^,^^5^ which frequently remains asymptomatic or presents with atypical symptoms. Therefore, CAD evaluation is a mandatory component of pre-LT assessment. In patients at elevated cardiovascular (CV) risk—such as those with metabolic dysfunction-associated steatohepatitis (MASH) or ≥2 traditional CV risk factors—the American Heart Association recommends the use of coronary computed tomography angiography (CTA).^6^^,^^7^ However, no global consensus currently exists on the optimal cardiac assessment prior to LT, and only around 30% of liver transplant centers in the United States routinely incorporate coronary CTA^8^ into their imaging protocols aside other tools.^9^

Electrocardiography (ECG), echocardiography, and single-photon emission computed tomography (SPECT) are commonly used in the preoperative workup,^7^^,^^10^^,^^11^ but these modalities show limited sensitivity and specificity for CAD detection in LT candidates.^12^^,^^13^ In patients over 40 years old or with limited physical activity but no known risk factors, pharmacological stress testing—such as dobutamine stress echocardiography (DSE) is often employed.^7^ However, patients with end-stage liver disease (ESLD) frequently present multiple comorbidities, including autonomic dysfunction, impaired chronotropic response, and reduced cardiopulmonary reserve, all of which limit the diagnostic performance of both exercise-based and pharmacologic stress tests.^14^, ^15^, ^16^, ^17^, ^18^, ^19^ Furthermore, the diagnostic accuracy of DSE is suboptimal in this population, with particularly low sensitivity in detecting CAD.^12^^,^^14^^,^^20^^,^^21^

Invasive coronary angiography (ICA) is considered as the reference standard in detecting coronary artery stenosis.^22^ Nevertheless, its use in ESLD patients is limited due to the risk of bleeding from coagulopathy^20^ and the potential for contrast-induced nephropathy, with up to 5.5% experiencing transient renal impairment after ICA.^22^ As a result, the optimal approach for cardiac risk stratification in the pre-LT setting remains debated.

More recently, coronary artery calcium (CAC) scoring has been proposed as a noninvasive method for CV risk stratification prior to LT, including in the 2021 guidelines.^23^ However, CAC scoring does not detect noncalcified plaque (NCP) and lacks the ability of graduating coronary stenosis severity.

Coronary CTA is a noninvasive imaging modality for a comprehensive assessment of coronary artery disease,^24^ capable of determining the degree of coronary stenosis and enables quantification of plaque burden and a characterization of atherosclerotic plaque composition—by distinguishing noncalcified lesions from calcified lesions—which aids in risk stratification and clinical decision-making. Additionally, the identification of “high-risk plaques” associated with increased Major Adverse Cardiovascular Events (MACE) risk^25^, ^26^, ^27^ is feasible by CTA. Thus, coronary CTA meets the necessary requirements to serve as an optimal noninvasive tool for pre-LT CV evaluation; however, literature is controversial—and some studies indicate that CAC alone may be sufficient for CV risk stratification prior to LT.^28^

This study aimed to assess the prognostic utility of both CAC and coronary CTA in ESLD patients undergoing LT for long-term outcomes.

## METHODS

### Study Design and Population

The study design of this single-center study was retrospective observational. Patients with ESLD due to different underlying liver diseases (MASH, MASH-LD, viral hepatitis, hemochromatosis and others presented at Table 1) listed for LT and referred to cardiac computed tomography (CT) for preoperative risk stratification were enrolled between March 2005 and December 2016. The study was performed according to the Declaration of Helsinki. An institutional review board approval was obtained.

**Table 1:** Demographic Data and Cardiovascular Risk Factors of Patients Undergoing Liver Transplantation (LT).

Age (years)	61 ± 8.5
Women, n (%)	56 (20.7 %)
BMI (kg/m^2^)	26.4 (range, 15.7–43.1)
Cardiovascular risk factors (CVRF)	
HTN	136 (50.4 %)
Smoking	23 (8.5%)
Dyslipidemia	53 (16.6 %)
Hypercholesterolemia	14 (5,2 %)
Diabetes	102 (37.8 %)
Positive family history	1 (0.4 %)
Atrial fibrillation	22 (8.1 %)
Underlying liver disease	
MASH-LD with cirrhosis	122 (45.2 %)
Viral hepatitis	
Hepatitis B	15 (5.5%)
Hepatitis C	61 (22.6 %)
Hepatitis D	2 (0.7 %)
Hepatitis E	1 (0.4%)
MASH	7 (2.6 %)
PBC	15 (5.5 %)
PSC	4 (1.5 %)
HCC	14 (5.2 %)
CCC	2 (0.7 %)
Cryptogenic liver cirrhosis	16 (5.9 %)
Wilson’s disease	2 (0.7 %)
Cystic liver disease	4 (5.9 %)
Hemochromatosis	2 (0.7 %)
Autoimmune hepatitis	4 (1.5 %)
Alpha 1 antitrypsin deficiency	1 (0.4 %)
Acute liver failure	2 (0.7 %)
Alcohol-related liver disease (ALD)	3 (1.1 %)
Vanishing bile duct syndrome	1 (0.4 %)
Liver abscess (+MASH-LD)	1 (0.4 %)
**MELD score (range)**	15.2 ± 6.5 (range: 6–40)
< 10	52 (19.2 %)
10–19	162 (60 %)
20–29	48 (17.8 %)
>30s	8 (3 %)

Abbreviations: MASH = metabolic dysfunction-associated steatohepatitis, PBC = primary biliary cirrhosis, PSC = primary sclerosing cholangitis, HCC = hepatocellular carcinoma, CCC = cholangiocellular carcinoma. HTN = arterial hypertension. BMI = body mass index.

Pre-LT CV evaluation included assessment of CV risk factors (pre-existing diabetes, arterial hypertension, cigarette smoking, lipid status, family history and body mass index), physical examination, electrocardiography and transthoracic rest echocardiography.

As part of our internal cardiac CTA study protocol.

• After the CAC score, CTA was appended only if 1) no severe renal dysfunction eGFR <45 ml/min/1.73 m^2^ was present, 2) heart rate target reached (<80bpm if 128-dual-source CTA and <65bpm if 64 –slice CTA) and if the 3) CAC score was not excessively high (>1000 AU). If CAD was above 1000 AU, ICA or myocardial stress testing was performed.
• Patients with suspected >50% stenosis by CTA (Coronary Artery Disease—Reporting and Data System [CAD-RADS] 3 and 4) were referred to either a myocardial perfusion test, or ICA, if possible.

#### Inclusion Criteria

Patients >35 years and with no or >1 risk factors (diabetes, severe arterial hypertension, peripheral artery disease, obesity (defined as body mass index >30 kg/m^2^), dyslipidemia, myocardial infarction (MI) signs in ECG, positive family history, smoking) and unknown CAD were referred to cardiac CT.

High-risk patients presenting several ( ≥ 3) risk factors and suspected myocardial ischemia based on symptoms and stress testing were primarily referred to ICA pre-LT and not considered for referral to CTA according to our internal in-hospital triage.

#### Exclusion Criteria

Prior MI or acute coronary syndrome (ACS), prior myocardial revascularization with percutaneous coronary intervention (PCI) or coronary artery bypass grafting (CABG), and patients who were listed for LT but finally did not undergo LT were the exclusion criteria.

### Coronary Computed Tomography Angiography

Non-contrast ECG-gated CAC CT with standardized scan parameters (detector collimation 64 × 1.5 mm; 120 kV) was performed, and the Agatston score was calculated. Then, coronary CTA was appended using either a 64-slice CTA (Sensation 64, Siemens, GE lightspeed and GE lightspeed VCT, GE - General Electric Company, USA; until December 2009) or 128-slice dual-source CTA (Definition FLASH, Siemens) with a detector collimation of 2 × 64 × 0.6 mm or 64 × 0.6 mm and a rotation time of 0.28 or 0.33 s, respectively. In patients examined with 64-slice CT, retrospective ECG gating was applied. In patients undergoing 128-slice dual-source CT, prospective ECG triggering was used in regular heart rates of <65 bpm (diastolic phase and 70% of cardiac cycle) and >65 bpm (systolic phase and 40% RR interval± 5% pending on heart rate), and retrospective ECG gating was used in patients with arrhythmia.

An iodine contrast agent (Ultravist 370 (Bayer Healthcare), Visipaque 320 (GE Healthcare) and Jopamiro 370 (Bracco Imaging)) was injected intravenously (flow rate of 3–6 mL/s + 40 cc saline chaser), triggered into an arterial phase (bolus tracking; 100 HU threshold; ascending aorta). Contrast volume varied between 65 and 120 ml according to the individual patient’s characteristics using a standardized scheme. Axial images were reconstructed with a 0.75 mm slice with (increment 0.4/medium-smooth kernel B26f).

Beta-blockers were administered for heart rate control (5 mg metaprolol i.v.) and the dose repeated after 3 min and the target heart rate was not achieved. Target heart rate was <80 bpm for dual-source CTA and <65 bpm for 64-slice CTA.

### CTA Image Analysis

Curved multiplanar reformation (cMPR) and oblique interactive MPR using 3D post-processing software (SyngoVia, Siemens Healthineers) were generated:

1) Coronary stenosis severity was scored visually according to the CAD-RADS^TM^^29^ score (0–5) as minimal^1^ <25%, mild^2^ 25–49.9%, moderate^3^ 50–69.9%, severe^4^ ≥70%–99% and^5^ occluded 100% on a per-coronary segment-base (AHA-modified-17-segment classification assisted by quantitative stenosis measurement using cMPR.
2) Coronary plaque phenotypes: High-risk plaque (HRP) analysis:
  • Low attenuation plaque was defined as hypoattenuating lesion with <150 HU. CT-density was screened with the “pixel lens” and the lowest HU recorded.^30^ LAP<30HU was defined as lipid-rich necrotic core^31^ and LAP<60 HU as fibrofatty.
  • An outer high-density rim with an inner hypodense area^32^ on images was defined as “Napkin-ring sign” because of its resembling appearance with a Napkin ring.
  • Spotty calcification was defined as a calcification of less than 3 mm size.
  • Positive remodeling was defined as a remodeling index of >1.1.^33^

A HRP was defined if a minimum of two criteria were present, and if at least one LAP <30HU or LAP <60 HU was present per patient.

3) Coronary plaque burden was calculated by summarizing the plaques per AHA coronary segment, and weighting for NCP component (plaque type 1 = calcified, 2 = mixed predominantly calcified, 3 = mixed predominantly noncalcified, 4 = noncalcified).

Image analysis was performed by two independent observers (>5 years and 10-year experience). Consensus reading was obtained.

#### Perioperative and Post Transplant Follow-up

All CV complications during LT were noted and any CV events (MI, onset of angina symptoms, arrhythmia and heart failure) were recorded within the early (in-patient setting) and late postoperative period (hospital discharge and last follow-up visit).

### Outcome Data Collection

Follow-up was performed via hospital chart results checkup. ICA results (stenosis % per coronary segment) and consecutive coronary revascularization procedures, either via PCI or CABG and ICA data, were collected.

Primary endpoint was all-cause mortality, and secondary endpoint acute MI defined as documented ACS, ST-elevation MI (STEMI), non-STEMI (NSTEMI) and/or by post-mortem histology of MI in case of death.^34^ Causes of death were recorded and stratified into all-cause and CV deaths.

### Statistical Analysis

Statistical analysis was performed using SSPS™ software (*V21.0, SPSS Inc., Chicago, USA*) and MedCalc (V12.5, Belgium). Quantitative variables are expressed as means ± standard deviation, and categorical variables are expressed as absolute values and percentages. A *P*-value of <0.05 was considered as significant. Normal distribution of data was tested with the Shapiro–Wilk and a histogram. Mean differences between parametric and normally distributed data were tested with the independent t-test and with Χ^2,^ or Fisher’s exact test for categorical data. Receiver operating characteristic (ROC) curves with area under the curves (AUCs) were generated for the CTA CAC, CAD-RADS + plaque burden for prediction of CV endpoints. Univariate Cox proportional hazards risk models were created for prediction of CV outcomes, and multivariate Kaplan–Meir analysis was performed.

## Results

**Figure 1:**
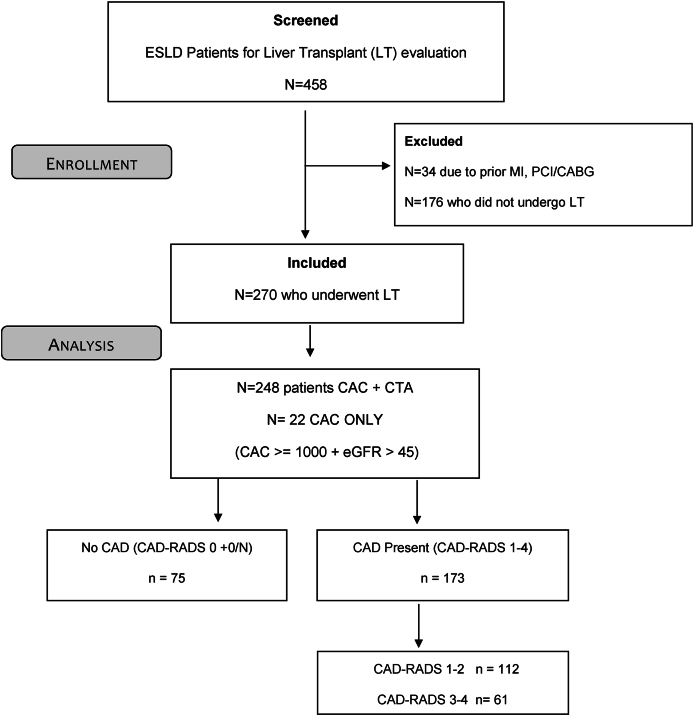
Patient flow chart.

The flow chart (Figure 1) illustrates patients’ recruitment. Of the 458 patients with ESLD screened who underwent coronary CTA for pre-LT assessment, 34 patients with prior ACS/MI or PCI were excluded, and 176 did not undergo LT. Finally, 270 (59%) who underwent LT at our center were included.

The baseline characteristics of the study cohort, including underlying liver diseases, are presented at Table 1. The mean age of the population was 61 ± 8.5 years. The majority of patients were men (79.3%). The Model for End-Stage Liver Disease (MELD score) was a mean of 15.2 ± 6.5 (range, 6–39.33). Follow-up was a mean of 7.5 ± 3.1 years (range, 2–13 years).

### Cardiac CT Results and CAC Score

Eighty-seven of 270 patients (32.2%) had CAC 0 AU, while 60 (22.2 %) had a CAC >300 AU. The mean CAC was 335.6 AU ±868.9 AU.

Two hundred forty-eight of 270 patients (91.6 %) underwent coronary CTA after the CAC score. In 22 patients, only the CAC score was available. The CTA was either not performed because they met the exclusion criteria and underwent either the CAC score only due to high CAC >1000 AU or renal dysfunction (Glomerural Filtration Rate (GFR) <45 kg/m^2^) or they were excluded due to poor image quality.

### Coronary CTA

**Figure 2:**
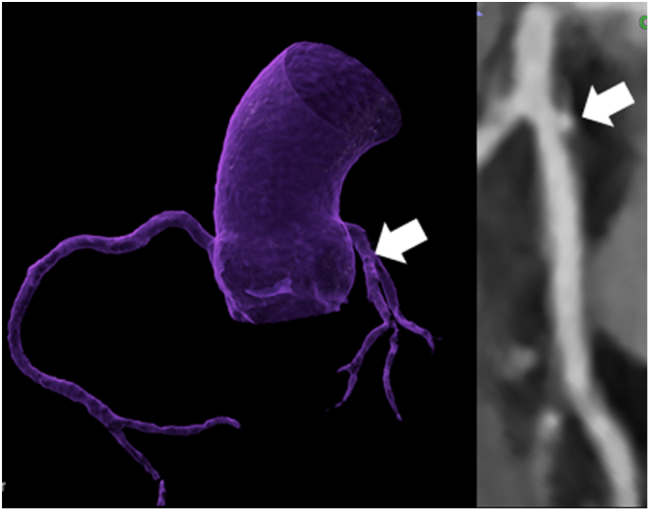
59-year-old male who underwent CTA pre-LT, mixed (predominantly noncalcified) plaque in the proximal and mid LAD with the napkin-ring sign (NRS) with recurrence of hepatitis C–associated liver cirrhosis prior to liver transplantation, MELD Score 21, metabolic syndrome with diabetes, obesity (BMI 32.4 kg/m^2^) and with 9 years survival after LT. CTA = computed tomography angiography; LAD, left anterior descending; LT = liver transplantation.

**Figure 3:**
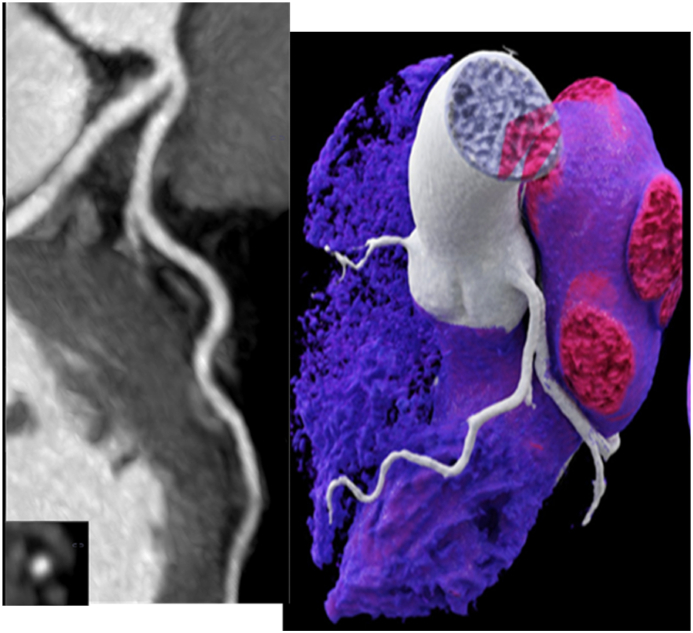
62-year-old female with hepatitis C, MELD Score 13, Child II stage, no alcohol since 30 years with post transfusion HCV after an accident, non-smoker since 34 years and no other risk factors. CTA ruled out coronary artery disease. LT was successful and outcome was excellent over 10 years. The 3DVRT (right) shows normal coronary arteries. CTA shows left anterior descending and circumflex artery (CX) (left, curved multiplanar reformation (cMPR)). CTA = computed tomography angiography; HCV = hepatitis C virus; LT = liver transplantation.

The majority (172/248; 69.4%) had CAD by CTA, defined as the presence of any plaque (CAD-RADS 1–5) and 75/248 (30.6%) had no CAD (no plaque, CAD-RADS 0). Among 248 patients undergoing CTA, the majority (n = 173, 69.8%) had CAD by CTA (CAD-RADS 1–5), and n = 75 (30.2%) had no CAD. Stenosis severity was minimal-to-mild (<50%) in 112 (45.1%), intermediate (50–70%) in 44 (17.7%), and severe (>70%) in 17 (6.5%) patients. Figure 2, Figure 3 show case examples.

Among patients with a CAC score of 0 AU, 13 (5.2%) had NCP on coronary CTA. HRP features **(**LAP <30 HU or LAP <60 HU with positive remodeling) including the napkin-ring sign (NRS) were present in 41 patients without MI and in 1 patient with MI (total prev. 42/248, 17%) (Table 3).

### Primary Endpoint

**Figure 4:**
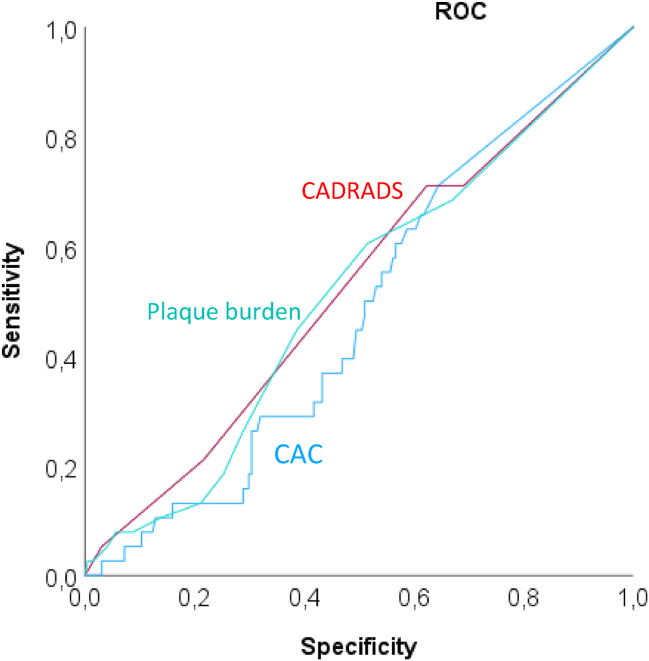
**ROC for prediction of the primary endpoint all-cause mortality:** Coronary CTA parameters showed a higher accuracy for coronary stenosis severity (CAD-RADS, red) c = 0.525 ± 0.051 (95% 0.425–0.625) and plaque burden (green) c = 0.513 ± 0.050 (95% CI: 0.415–0.611) compared to coronary artery calcium (CAC) (blue) c = 0.480 ± 0.470 (95% CI: 0.388–0.572). **Abbreviations:** CAC = coronary artery calcium score; CI = confidence interval; ROC = receiver operating curve; SD = standard deviation.

All-cause mortality rate was 46/270 (17 %). The majority of deaths, 43 (93.5 %) were non-cardiac and three (6.5%) CV deaths (one patient with MI and two patients with cardiopulmonary failure) occurred. Causes of death are shown in Table 2. On ROC analysis, the discriminatory value of the CTA parameters CAD-RADS and plaque burden was poor with c = 0.525 ± 0.051 (95% 0.425–0.625) for CAD-RADs and c = 0.513 ± 0.050 (95% confidence interval [CI]: 0.415–0.611) for plaque burden and slightly higher than for CAC with c = 0.480 ± 0.470 (95% CI: 0.388–0.572) (Figure 4a). Coronary stenosis severity (CAD-RADS) was significantly associated with mortality on Kaplan–Meier analysis (*P* < 0.001).

**Table 2:** All-cause Mortality: Causes of Death in LT Patients (n = 270).

Causes of death	n = 46
Multiorgan failure	9 (19.6 %)
Sepsis	16 (34.8 %)
Liver failure	4 (8.7 %)
Cardiac and pulmonary failure	2 (4.3 %)
Hepatitis	2 (4.3 %)
Myocardial infarction	1 (2.2 %)
Graft versus host disease	1 (2.2 %)
Advanced cancer	1 (2.2 %)
Intracerebral hemorrhage	2 (4.3 %)
Posttransplant lymphoma	1 (2.2 %)
Recurrent HCC	2 (4.3 %)
Sudden death (unclear)	1 (2.2 %)
Unclear	2 (4.3 %)
Aspiration of blood	1 (2.2 %)
Respiratory insufficiency	2 (4.3 %)

Abbreviations: HCC = Hepatocellular Carcinoma.

**Table 3:** CTA Stenosis Severity (CAD-RADS) in LT Patients (n = 270) and Their Correlation With Secondary Endpoint Myocardial Infarct (MI): ACS-STEMI or NSTEMI.

	No event	MI
	264/270 (97.8 %)	6/270 (2.2 %)
CAD-RADS 0	75 (30.2 %)	0 (0 %)
CAD-RADS 1	14 (5.2%)	0 (0 %)
CAD-RADS 2	97 (35.9 %)	1 (0.37 %)
CAD-RADS 3	40 (14.8%)	4 (1.48%)
CAD-RADS 4 + 4/N	9 (3.33 %) +7 (2.6%)	1 (0.37 %)
CAD-RADS 5	0 (0 %)	0 (0%)
No CTA, only CAC	22 (8.1 %)	0 (0 %)

CAD-RADS = coronary artery disease-reporting and data system

CAD-RADS N = non-diagnostic image quality. If calcified plaque was present and stenosis >50% and >70% could not be ruled out due to blurring artifacts - > managed as CAD-RADS 4/N.

CAD-RADS 0-no stenosis, CAD-RADS 1 (<25 % stenosis), CAD-RADS 2 (25–49% stenosis), CAD-RADS 3 (50–69% stenosis), CAD-RADS 4 (70–99% stenosis), CAD-RADS 5 (100 %) total occlusion.

CAC (categorized in CAC 0, CAC 1–10, CAC 11–100, CAC 101–400, and CAC >400 AU) was not predictive for both endpoints’ mortality and MI (*P* = 0.76, *P* = 0.29).

### Univariate Cox Regression

Age was not associated with mortality (hazard ratio [HR]: 0.988), and the relative risk for CAD-RADS was HR: 1.125 and for CAC HR: 1.0. On multivariate Cox regression, plaque burden was associated with all-cause mortality (HR: 1.1, *P* = 0.029; 95% CI: 1.0–1.6), while CAC score did not.

### Secondary Endpoint (MI)

There were 6 MI (3 STEMI, 3 NSTEMI). MI rate was 0% in CAD-RADS 0–1 and 5, 1 MI occurred in CAD-RADS 2 and 4 (16.7 %) and 4 events in CAD-RADS 3 (66.7 %), Table 2. Time to MI was mean 4.7 years (range, 1.6–9.5 years). All MI occurred after LT.

Table 3 shows coronary stenosis severity in patients with and without MI. On ROC, AUCs were consistently higher for the secondary than for the primary endpoint (CAD-RADS: c = 0.714 ± 0.099, *P* = 0.074; 95% CI 0.520–0.908 and for plaque burden (G-score) c = 0.746 ± 0.073 (95% CI 0.604–0.889). On univariate Cox regression, the HR for CT parameters were: CAC: HR: 1.00 (95%CI: 0.999–1,00), *P* = 0.682, CAD-RADS: HR: 1.793 (95% CI: 0.791–4.065), *P* = 0.162, plaque burden (G-score): HR: 1.163 (95% CI: 0.940–1.443, *P* = 0.163). Age was not associated (HR: 1.004, 95% CI: 0.898–1.122, *P* = 0.946) with MI.

There were 5 MI events after ICA, in 3 patients with CTA >50% stenosis and n = 2 MI events in patients with CTA <50 % stenosis. Invasive coronary angiograph (ICA) rate after CTA was 10 % (27 of 270 patients), and 10 of 27 had >50% stenosis (n = 7 underwent CABG, n = 3 PCI). Table 4 shows ICA results after CTA, according to coronary stenosis severity (CAD-RADS). In six of 27 patients who underwent ICA with CAD-RADS 3, five showed stenosis >50% on CABG and only one stenosis <50 % in ICA (see Table 5).

**Table 4:** Plaque Types by Coronary CTA in LT Patients (n = 270) With and Without Secondary Endpoint MI (ACS-STEMI or NSTEMI).

	total (n = 270)	MI (n = 6)
1 calcified plaque	117 (43.3 %)	4 (66.7 %)
2 mixed plaque (CP > NCP)	19 (7 %)	0 (0 %)
3 mixed plaque (NCP > CP)	6 (2.2 %)	1 (16.7 %)
4 noncalcified plaque	8 (3 %)	0 (0 %)
5 HRP (with NRS)	41 (15.2 %)	1 (16.7 %)
6 only CAC	22 (8.1 %)	0 (0 %)

Abbreviations: ACS = acute coronary syndrome; CAC = coronary artery calcium score; CP = calcified plaque; HRP, high-risk plaque; LT = liver transplantation; MI = myocardial infarction; NCP = noncalcified plaque. NSTEMI = non-STEMI; NRS= napkin-ring sign. STEMI = ST-elevation myocardial infarction. CAD-RADS N = poor image quality, non-diagnostic.

**Table 5:** Coronary Artery Stenosis by CTA (CAD-RADS) and Invasive Coronary Angiography (ICA) Results (n = 27).

	CTA		ICA
	**CAD-RADS 0**	0/27	<50 % stenosis: 0
			>50 % stenosis: 0
	**CAD-RADS 1**	1/27	<50 % stenosis: 0
			>50% stenosis: 1
	**CAD-RADS 2**	16/27	<50 % stenosis: 12
27 pts.			>50 % stenosis: 4
	**CAD-RADS 3**	6/27	<50 % stenosis: 1
			>50 % stenosis: 5
	**CAD-RADS 4**	1/27	<50 % stenosis: 1
			>50 % stenosis: 0
	**CAD-RADS 5**	0/27	<50 % stenosis: 0
			>50 % stenosis: 0
	**CAD-RADS N**^a^	3/27	<50 % stenosis: 3
			>50 % stenosis: 0

CTA = computed tomography angiography.

a N = non-diagnostic image quality of CTA; CAC score was used for patient management. Ff calcified plaque was present visually not allowing to rule out stenosis >50% or >70%, ICA was performed - if possible.

The mean waiting time for all 270 patients included in this study, to receive a suitable liver allograft was 9 months, and ranging from one to 24 months.

## Discussion

Cardiovascular disease is among the leading three causes of morbidity and mortality following liver transplantation.^35^ Cardiac risk stratification in patients awaiting liver transplantation remains controversial.^36^ Our study supports the use of coronary CTA as a safe and effective non-invasive tool in the pre-liver transplantation (LT) setting, allowing for the exclusion of significant coronary artery stenosis and improved CV risk stratification. Over a long-term follow-up (mean 7.5 years), a low overall event rate was observed, and notably, a 0% MI rate in patients with negative CTA findings or minimal CAD (CAD-RADS 1). Myocardial infarction (MI) occurred only in those with intermediate or severe coronary stenosis (CAD-RADS 3–4), and in just one event in a patient with non-obstructive CAD (CAD-RADS 2).

To our knowledge, this is the first long-term outcome study using CTA in this population with a mean follow-up exceeding 7 years. Our findings also support the usefulness of coronary CTA in pre-LT CV risk stratification.

In our cohort, coronary stenosis and total plaque burden (weighted for noncalcified components) assessed by CTA were predictors of all-cause mortality, whereas the CAC score was not. Our results align with a recent study by Rodrigues *et al.* (2024)^37^ which found that CAD-RADS ≥3, but not CAC scores, predicted MACE over a 4-year median follow-up (HR: 5.8, 95% CI: 1.6–20.6, *P* = 0.006) in 157 patients. Our study not only confirms these findings but also extends them, benefiting from a larger sample size and longer follow-up period. While other studies support the use of coronary CTA for CV risk stratification in patients undergoing liver transplantation (LT), few have specifically focused on the ESLD population. Koshy *et al.* (2022) (15) reported accelerated coronary atherosclerosis progression in LT recipients, particularly in those with HRP features, highlighting the prognostic value of plaque morphology analysis. Cassagneau *et al.*^38^ and Poulin *et al.*^8^ found similar results in smaller cohorts, but without detailed analysis of plaque burden HRP. In contrast, our study uniquely evaluated HRP features, which are emerging as powerful imaging biomarkers with a reported 4-fold increase in MACE risk,^31^ and the prevalence of HRP features indicating lipid-necrotic core lesions in our cohort with 17% was considerable. Notably, even 5.2% of our patients with CAC 0 had NCP. NCP burden (defined as mixed fibro-fatty) is another important predictor of CV events.^27^

CAD prevalence in ESLD patients remains variably reported, with ICA studies suggesting a prevalence ranging from 2.5% to 27%^20^^,^^39^^,^^40^ for obstructive disease (>50% stenosis). Our results indicate a higher prevalence of coronary atherosclerosis overall when including non-obstructive disease detected by CTA, although obstructive CAD (>50% stenosis) was also observed in about 25% of cases—which is clinically relevant given the risk of adverse outcome of the LT procedure. Severe stenosis can increase intraoperative risk for myocardial ischemia, and additional anatomical risk factors such as left ventricular outflow tract obstruction—often due to basal septal hypertrophy—can also be accurately assessed by CTA.^7^ However, medical therapy of non-obstructive CAD (e.g. with statins), is also important in LT patients, to prevent further cardiac events and progression of CAD.

However, it is still a matter of controversy which tests should be used to predict the CV risk of LT candidates.^6^ Three liver transplant–specific CV risk scores have been proposed so far. The CAD-LT score^41^ estimates pre-LT risk of significant CADbut is limited by its focus on CAD alone, which represents a small fraction of post-transplant CV events. The Cardiac Arrest Risk Index (CARI score)^42^ identifies candidates at risk for early post-LT arrhythmias. The Cardiovascular Risk in Orthotopic Liver Transplantation (CAR-OLT score)^43^ based on clinical variables, aims to predict 1-year MACE without relying on stress testing.

Optimal pre-LT CV assessment is still under constant investigation. CAC scoring has recently been proposed as a noninvasive screening tool for CAD in this setting.^44^ Pagano *et al.*.^45^ demonstrated that a CAC <100 AU was associated with a low prevalence of obstructive CAD, while patients with scores ≥400 AU exhibited a significantly higher burden of atherosclerosis and increased risk of posttransplant CV events.

In this study, we analyzed the efficacy of CTA and CAC scoring as a reliable noninvasive tool to determine the prevalence of CAD in patients assessed for LT and to predict perioperative and postoperative CV events in patients undergoing LT. Several publications have evaluated the potential of CTA to detect obstructive CAD^46^; however, it is not fully clear whether the CAC score is sufficient enough, or CTA would be more accurate.

The strength of CTA^47^ lies in its high diagnostic accuracy for the detection of obstructive coronary artery disease. A meta-analysis reported that coronary CTA has a sensitivity of 95–99% and a specificity of 68–93% for detecting significant CAD, demonstrating higher diagnostic performance than exercise ECG or SPECT.^48^

The advantages of CTA include its noninvasiveness, which is especially important in many ESLD patients due to their higher bleeding risk during invasive procedures and a comprehensive assessment of coronary artery stenosis and atherosclerotic plaque burden. Our findings are consistent with the recent data using AI-driven quantitative CTA (AI-QCT) for quantification of total plaque volume (TPV) revealing a 56% increased risk of CV events for TPV (adjusted HR: 1.56; *P* = 0.001). The integration of TPV into conventional risk models significantly improved the discrimination of adverse outcomes.^49^ Novel AI-driven technology nowadays allows for an even more accurate and nuanced quantification of total plaque burden and components (noncalcified vs. calcified).^49^

On ROC, CAD-RADS and plaque burden showed a higher accuracy than the CAC score, with an overall moderate discriminatory power for all-cause mortality, likely explained by the fact that the majority of deaths were noncardiac and related to our patient’s specific conditions (liver disease). Furthermore, only 3 of 46 deaths (6.5%) were CV in origin, and the prevalence of MI (secondary endpoint) was low with only 6 MI. However, on the other hand, the low number of events proofs safety of coronary CTA as a valuable imaging modality in patients prior to liver transplantation for CV risk stratification.

Furthermore, the absolute c-values of all coronary CTA parameters for prediction of mortality were lower than in studies in which mortality was mainly CV. This is explained by the fact that most of our patients died due to their underlying ESLD and related complications such as sepsis and organ failure—but not from a cardiac event. Therefore, results of the secondary endpoint differ in LT patients compared to otherwise healthy patients with suspected coronary heart disease, which are usually referred to coronary CTA.

However, for secondary endpoint MI, the AUC was consistently higher for coronary CTA parameters (stenosis severity and plaque burden), highlighting the excellent discriminatory value of CTA for the prediction of the more specific CV endpoint ACS (c = 0.714 – c = 0.746). Whether obstructive CAD in LT patients is as highly relevant for prediction of mortality as in otherwise healthy subjects has been debated controversially recently in an opinion letter.^50^

CAC did not predict both primary and secondary endpoints in our cohort, reaffirming the known limitations of CAC as a stand-alone marker for risk assessment. It is well-known that a high CAC of >300 Agatston Units (AU) improves CV risk stratification as compared to the Framingham Risk Score^51^ in asymptomatic patients without liver disease, while the prevalence of high CAC >300 AU was rather low in our cohort with 22.2 % due to the fact that as part of our protocol, patients with very high CAC scores (>1000 AU) were not referred for CTA to avoid false positive findings by CTA. Instead, CV assessment was performed using ICA (or myocardial perfusion imaging).

Furthermore, importantly, we implemented a specific in-hospital triage scheme and included only those with low-intermediate likelihood of CAD. High-risk patients and those with known CAD were directly referred to ICA—leading to an overall low MI rate after LT and excellent outcomes with few CV deaths only. Accordingly, our triage system can be regarded as “safe” to prevent adverse cardiac events. This unique feature of our cohort introduces a selection bias toward low-intermediate risk patients and may also explain why MELD scores were lower as in LT-recipient cohorts including high-risk patients.

Finally, of note, DSE^9^ is also a valuable alternative noninvasive imaging modality for assessment of CAD in LT-recipients—if available. Furthermore, myocardial stress perfusion imaging is rather recommended in elderly >65 years according to the AHA Chest Pain Guidelines in patients with suspected CAD and intermediate likelihood, and coronary CTA in those <65 years^52^—due to the high likelihood of high calcium scores in elderly> 65 years, limiting the accuracy of coronary CTA (but not the accuracy of myocardial perfusion imaging).^53^

### Study Limitation

This was a retrospective, single-center study with inherent selection bias. CTA was not performed in all patients due to high CAC scores>1000 AU; therefore, patients at the highest risk were excluded from coronary CTA. Additionally, the low number of MI events (n = 6) limits the statistical power for secondary endpoint MI. Finally, we used the visual scoring of plaque burden (segment involvement score (SIS) and G-score), which has a higher interobserver variability than the most recently introduced fully automated AI-QCT.^49^

Our findings indicate that coronary CTA is a safe and effective tool for long-term CV risk stratification in ESLD patients undergoing liver transplantation. It provides superior and more specific details over CAC scoring for prediction of all-cause mortality through the assessment of coronary stenosis and plaque burden.

## Clinical Perspective

Pre-transplant coronary CTA provides superior CV risk stratification compared to CAC scoring alone, with the added benefit of potentially reducing the need for invasive coronary angiography. By offering detailed assessment of stenosis severity, total plaque burden, and HRP features, CTA supports a more comprehensive and individualized risk evaluation. Its integration into standard pre-LT protocols could improve clinical decision-making and patient outcomes. Novel AI-driven technology may have the potential to further enhance CV risk stratification using CTA by allowing for a fully automated quantification of TPV and the components of atherosclerosis.

## DISCLOSURES

None.

## CREDIT AUTHORSHIP CONTRIBUTION STATEMENT

Pietro G. Lacaita: Writing – review & editing, Writing – original draft, Data curation, Visualization, Armin Finkenstedt: Writing – review & editing, Data curation, Thomas Senoner: Writing – review & editing, Methodology, Data curation, Heinz Zoller: Writing – review & editing, Data curation, ^,^ Guy Friedrich: Writing – review & editing, Data curation, Mathias Pamminger: Writing – review & editing, Data curation, Yannick Scharll: Writing – review & editing, Data curation, Gerlig Widmann: Writing – review & editing, Data curation, Gudrun M. Feuchtner: Writing – review & editing, Writing – original draft, Visualization, Software, Methodology, Investigation, Data curation, Conceptualization.

## FUNDING

None.

## DECLARATION OF COMPETING INTEREST

The authors declare that they have no known competing financial interests or personal relationships that could have appeared to influence the work reported in this paper.
